# In-DRAM Cache Management for Low Latency and Low Power 3D-Stacked DRAMs

**DOI:** 10.3390/mi10020124

**Published:** 2019-02-14

**Authors:** Ho Hyun Shin, Eui-Young Chung

**Affiliations:** 1 Samsung Electronics Company, Ltd., Hwasung 18448, Korea; hhshin@yonsei.ac.kr; 2 School of Electrical and Electronic Engineering, Yonsei University, Seoul 03722, Korea

**Keywords:** 3D-stacked, DRAM, in-DRAM cache, low-latency, low-power

## Abstract

Recently, 3D-stacked dynamic random access memory (DRAM) has become a promising solution for ultra-high capacity and high-bandwidth memory implementations. However, it also suffers from memory wall problems due to long latency, such as with typical 2D-DRAMs. Although there are various cache management techniques and latency hiding schemes to reduce DRAM access time, in a high-performance system using high-capacity 3D-stacked DRAM, it is ultimately essential to reduce the latency of the DRAM itself. To solve this problem, various asymmetric in-DRAM cache structures have recently been proposed, which are more attractive for high-capacity DRAMs because they can be implemented at a lower cost in 3D-stacked DRAMs. However, most research mainly focuses on the architecture of the in-DRAM cache itself and does not pay much attention to proper management methods. In this paper, we propose two new management algorithms for the in-DRAM caches to achieve a low-latency and low-power 3D-stacked DRAM device. Through the computing system simulation, we demonstrate the improvement of energy delay product up to 67%.

## 1. Introduction

**Figure 1:**
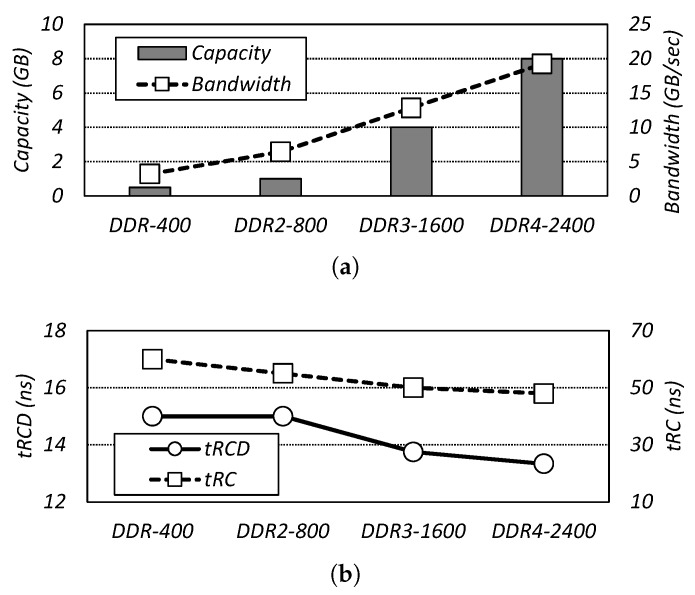
Comparison of dynamic random access memory (DRAM) capacity, bandwidth, and latency improvement by DRAM generation [4,5,6,7]. (**a**) Capacity and bandwidth of DRAM. (**b**) DRAM access latency: row address to column address delay (*tRCD*) and row cycle time (*tRC*).

The latency of dynamic random access memory (DRAM) has been a critical issue for two primary reasons [1]. Firstly, while the processing speed of central processing unit (CPU) has been continuously improved, DRAM latency has remained relatively unchanged for decades. This speed gap, called the memory wall, causes significant bottlenecks in the overall computing performance [2,3]. As shown in Figure 1a, while the capacity and bandwidth have increased 16 and 6 times over time, respectively, the timing constraints representing the DRAM latency, row address to column address delay (*tRCD*) and row cycle time (*tRC*), have only been improved by 11.2% and 20.0%, respectively [4,5,6,7].

Secondly, the processing speed of big data workloads is affected by the memory latency, as well as bandwidth. Russell et al. proved that the instructions per cycle of the applications dealing with big data could be significantly improved by reducing the DRAM latency [8]. This is because the data stream of big data is likely to have large dependency between its elements. In particular, on-line transaction processing (OLTP), which supports high transaction-oriented applications, is a representative example of latency-sensitive applications [9]. In addition, recent AI applications require large amounts of memory to handle large amounts of data, and require low latency to provide real-time data processing. In other words, we expect to see an increasing number of applications that simultaneously demand high capacity and low latency.

DRAM devices are being transformed into various structures as a result of recent developments in die stacking through silicon via (TSV) [10]. For example, the die stacking of homogeneous DRAM chips extends their capacity without power and performance losses [11,12]. Moreover, a heterogeneous combination of logic and DRAM dies, such as for a high-bandwidth memory (HBM) or hybrid memory cube (HMC), increases the data bandwidth without a significant power overhead [13,14]. The meaning of the power implied above is precisely the power relative to the performance value, such as capacity and bandwidth. For example, when comparing Graphic Double Data Rate 5 (GDDR5) and HBM with the same capacity and bandwidth performance, HBM’s power consumption is significantly smaller. However, though they have enhanced the memory sub-system in terms of capacity and bandwidth, the latency improvements have been neglected.

In order to overcome the long latency problem of DRAM, many computers embed numerous caches in the CPU. The cache not only overcomes the long latency of DRAM, but it also provides data locality for the pre-fetched pages. Thus, it offers large bandwidth locally in a CPU. However, since a typical cache is implemented using static random access memory (SRAM), it incurs large costs and consumes a high amount of leakage power. As a result, it is essential to reduce the DRAM latency itself to improve memory access latency (In this paper, DRAM latency refers to the time required for a DRAM controller to read or write data to a DRAM device, and memory access latency represents the latency required to access the data of the cache or DRAM by the processor instructions.).

The in-DRAM cache, which is embedded in a DRAM device, has several unique characteristics that differ from the processor cache [15]. First, the cache itself is placed in the DRAM, but its operation is managed by the DRAM controller. This is because the interface between the controller and the DRAM follows the DRAM timing constraints specified in joint electron device engineering council (JEDEC), which maintains high compatibility with the current computing system. Of course, there are various ways to implement the in-DRAM cache and its manager, such as operating systems (OS) or processor modifications. However, such methods require many modifications to the current computing system, and eventually degrade compatibility. We designed the manager to the DRAM controller so that the proposed method could follow the JEDEC specification, and implemented the in-DRAM cache in the DRAM device. Secondly, the capacity of the in-DRAM cache increases proportionally to the DRAM capacity and is much larger than the processor cache. For example, when hundreds of gigabytes of DRAM are mounted in a system, while the memory capacity of the processor cache remains constant at several hundred megabytes, the capacity of the in-DRAM cache can be up to tens of gigabytes. However, this large-capacity in-DRAM cache requires a larger tag size. This results in long tag access latency, which in turn increases the overall memory access latency. To overcome this problem, the data transfer granularity between the DRAM and in-DRAM cache, which is called cache block size, must be increased. However, this causes significant power consumption.

Power issues in DRAMs are very important in terms of minimizing the energy consumed by the DRAM chip itself, and are also critical parameters for 3D-stacked DRAMs from a thermal point of view. Since a 3D-stacked DRAM chip consists of several dies, it is very difficult to emit the heat generated inside the chip to the outside. This heat degrades the retention characteristics of the DRAM cells, and thus DRAM requires a shorter refresh cycle. However, reducing the refresh cycle of the high-capacity 3D-stacked DRAM results in more heat, which causes the retention time of the DRAM cell to decrease again. Therefore, thermal problems in 3D-stacked DRAMs are very sensitive design parameters and must be overcome.

Considering various properties of the in-DRAM cache, this paper proposes two new in-DRAM cache management algorithms for the data replacement, particularly to maximize its efficiency and minimize its energy consumption. In addition, the proposed management algorithms are not tied to a specific in-DRAM cache architecture, and can be appropriately adapted to general architectures.

## 2. Background and In-Dynamic Random Access Memory (DRAM) Cache Architecture

**Figure 2:**
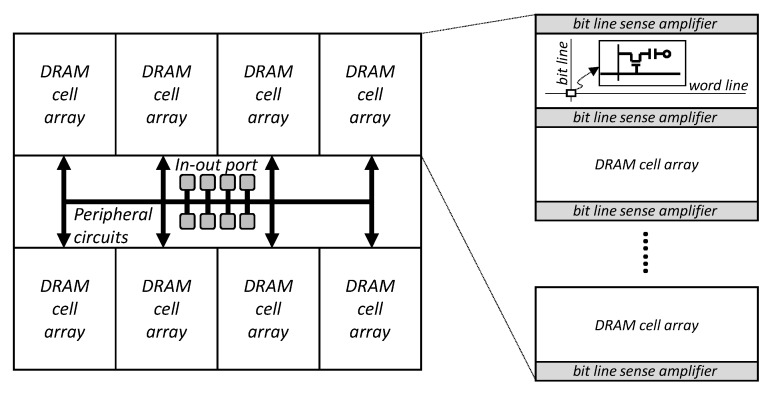
Conventional DRAM structure.

A DRAM chip consists of the DRAM cell array area and peripheral circuits, including several in-out ports (Figure 2). Here, the DRAM cell region is composed of a plurality of sub-arrays, including DRAM cells and bit-line sense amplifiers. As mentioned in Section 1, DRAM latency improvements are very slow, and there are many reasons for this. The reason for the slow latency improvement is directly related to cost and power consumption [16,17]. In order to reduce the sensing and pre-charge time, for example, the number of cells connected per bit-line should be reduced [18]. However, this leads to an increase in the number of bit-line sense amplifiers, and thus increases the chip size. Moreover, timing constraints, such as CAS latency (*tCL*) are mainly influenced by the speed of the data path. In order to improve this speed, the capacitive metal loading of the data path signal should be decreased, or its driver strength should be increased. However, these approaches may increase the cost or power consumption. Consequently, the latency of a DRAM device must be optimized with the simultaneous consideration of multiple side-effects. In this paper, we focus on the in-DRAM cache among various skills to reduce the latency of DRAM, and discuss its management method.

We deal with three types of in-DRAM cache structures based on recently published tiered-latency DRAM (TL-DRAM) and center high-aspect-ratio mats (CHARM) [19,20].

**Figure 3:**
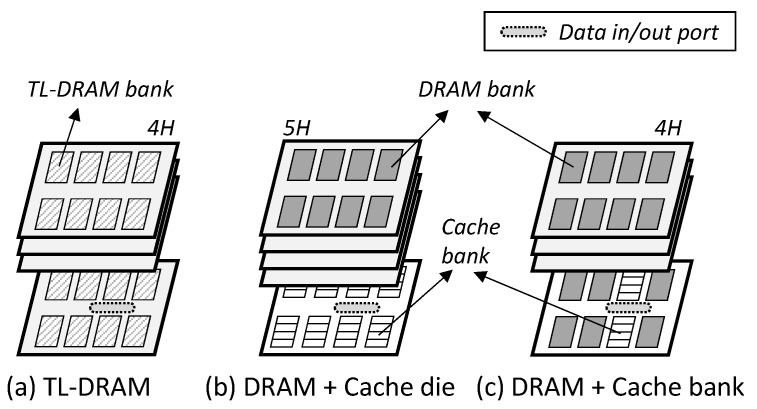
In-DRAM cache architectures.

- TL-DRAM: This divides the bit line of the DRAM array into two segments and uses the long one as the DRAM memory, and the short one as the in-DRAM cache [19,21]. Here, the TL-DRAM exploits the characteristic that the short bit line improves the sensing and the pre-charge speed, and uses it as a cache memory. Figure 3a shows the TL-DRAM architecture, which is the same in terms of the overall DRAM structure. However, the DRAM array belonging to one bank is different from the conventional one.
- Cache-die: This utilizes a single die among the 3D-stacked dies as the cache (Figure 3b). The in-DRAM cache can be implemented as SRAM or DRAM, but only the DRAM is covered in this paper. This architecture has the advantage of being able to implement a significant amount of cache capacity, but it has the disadvantage of requiring a large area overhead.
- Cache-bank: This is similar to the CHARM structure [20]. Some DRAM banks are used as low-latency DRAM caches, and this paper calls them cache banks (Figure 3c). It has a smaller cache capacity than the cache die, but it can significantly reduce the latency because the cache banks are close to the input/output interfaces of the DRAM.

In this work, we consider the three types of in-DRAM architecture described above at the same time. This is because the purpose of this paper is not to propose a new in-DRAM architecture, but to describe its efficient management algorithms. The cache replacement policy is also important. The most representative cache replacement algorithms are fist-in-first-out (FIFO) and least-recently-used (LRU). The FIFO policy removes the first block accessed the first time, regardless of how often or how many times the cache is accessed. Conversely, LRU discards the least recently used items first, and is a commonly used policy because it generally exhibits better hit-ratio characteristics. However, since it takes a long time to find the appropriate replacement items, it is not appropriate for in-DRAM caches that are very sensitive to latency. Therefore, we chose to adopt the FIFO policy as the default replacement policy for the in-DRAM cache due to its fast operating time. We tackle these issues in Section 3 and Section 4 in more detail.

## 3. Exploration of in-DRAM Cache Management

To design the in-DRAM cache and its management scheme, it is important to distinguish between the properties of the typical caches and the in-DRAM caches [1]. This is because in-DRAM cache management techniques are fundamentally based on the processor cache. This section describes the key design parameters of the in-DRAM cache that are distinct from the processor cache.

### 3.1. Trade-Off between Capacity and Latency

**Figure 4:**
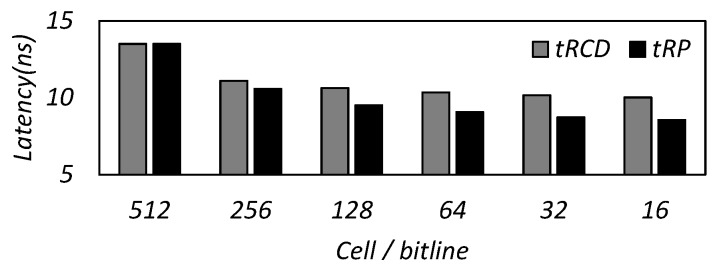
Changes in *tRCD* and *tRP* according to the various cells per bit line.

**Figure 5:**
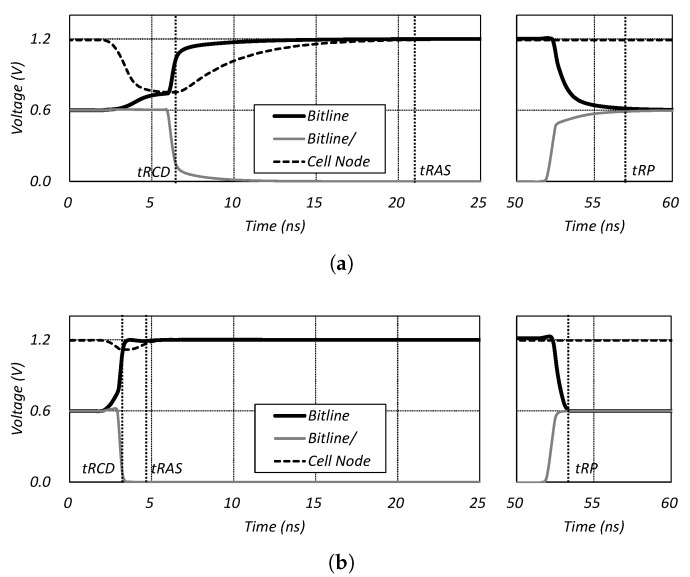
(**a**) SPICE simulation waveform with 512 cells per bit line, (**b**) Spice simulation waveform with 64 cells per a bit-line and pre-charge time.

The capacity of the in-DRAM cache is generally much larger than the processor cache. While the processor cache, which is implemented by SRAM, has limited capacity growth due to the power consumption and area overhead, since the in-DRAM cache is configured by DRAM cells, the capacity can be expanded at a low cost. However, the capacity of such an in-DRAM cache is in a trade-off relation with latency depending on how many cells are connected to a bit line. This is because as more cells are connected to one bit line, the capacity of the DRAM increases, while the sensing speed decreases. Figure 4 shows the simulation program with integrated circuit emphasis (SPICE) simulation results of the *tRCD* and *tRP* representing the sensing and pre-charge speed, respectively. The figure shows that when 64 cells are connected to a bit line, *tRCD* and *tRP* are set to saturation. In addition, Figure 5 shows the waveform of the bit line and cell node for the 512 and 64 cells per bit line. Based on these results, we assumed the 64 cells per bit line as the basic configuration of the in-DRAM cache.

### 3.2. Trade-Off between Tag Size and Power Consumption

The processor cache consists of data and tags in the CPU, which greatly reduces hundreds of nanoseconds of memory-access latency to tens of nanoseconds. Therefore, even though the size of the tag is large and its read-speed is somewhat slow, it is not a big deal on the overall memory access time. On the other hand, although the capacity of the in-DRAM cache is large and its hit ratio is thus quite high, the latency that can be reduced by the in-DRAM cache is only several nanoseconds. Therefore, it is very important to minimize the tag access time.

**Figure 6:**
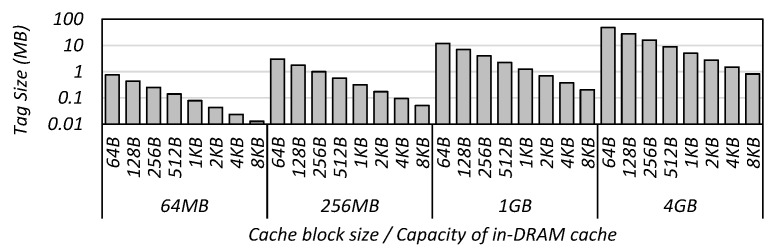
Tag-size variation with the block size and the capacity of in-DRAM cache.

The access time of the tag is influenced by the block size and the capacity of the in-DRAM cache. The larger the cache capacity or the smaller the cache block size, the larger the tag size. Figure 6 shows that the tag size grows from several KB to tens of MB depending on the block size and the capacity.

There are two ways to reduce the tag size. One is to reduce the capacity of the cache, and the other is to increase the cache block size. However, the former is not the ultimate goal of an in-DRAM cache. Therefore, we should increase the cache block size, which has other side-effects. Firstly, a cache block size which is too large can cause significant time overhead and power consumption for the data transfer. Secondly, for applications with low locality, it lowers the hit ratio of the in-DRAM cache. Therefore, it needs to design very sophisticated cache management techniques that considers these aspects.

## 4. Proposed In-DRAM Cache Management Algorithms

Typical DRAMs use a rank and bank interleaving policy to maximize data bandwidth. It maximizes the reuse rate of any pre-activated row address. This property motivated us to define the block size of the in-DRAM cache as the total data contained in a specific row address of all ranks and banks in the 3D-stacked DRAM. This method is disadvantageous in terms of time and power consumption, because a single data transfer operation moves hundreds of KB of data at the same time. On the other hand, it has the advantage that the tag access time can be reduced by minimizing the tag size. Therefore, it is important to maximize the hit ratio of the in-DRAM cache and to minimize the performance and power damage caused by the transfer. We discuss how to effectively utilize the in-DRAM cache by proposing two new in-DRAM cache management algorithms in the sections below.

### 4.1. Critical Data Detection and Evaluation Scheme

**Figure 7:**
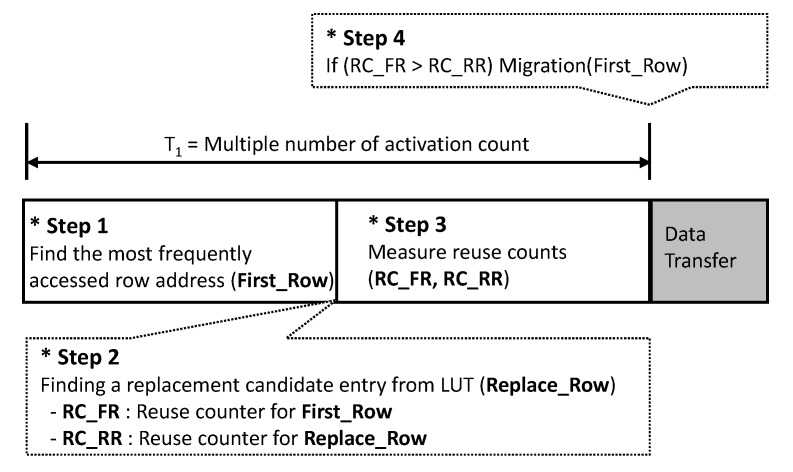
Descriptions of the proposed Critical Data Detection and Evaluation (CDDE) algorithm.

The Critical Data Detection and Evaluation (CDDE) scheme is designed to maximize the hit ratio of an in-DRAM cache. This is a technique that evaluates and replaces the criticality of new data, rather than replacing it with new data unconditionally when a cache miss occurs. Therefore, the proposed technique is divided into the critical data detection stage and evaluation stage. Figure 7 shows the brief description of the proposed algorithm. A unit cycle to determine a data transfer is defined by multiple activation counts, called *T*${}_{1}$. *T*${}_{1}$ is divided into four steps, as shown below.

- Step 1: The algorithm finds the most frequently accessed row address (*First_Row*).
- Step 2: The in-DRAM cache manager selects a candidate entry (*Replace_Row*) to be replaced in the tag, where the replacement policy can be the least recently used (LRU) or first-in first-out (FIFO) that are similar to the legacy replacement policy [1]. In this paper, we use the FIFO, which can minimize the time delay for the candidate selection.
- Step 3: It measures the reuse counts for the *First_Row* and *Replace_Row*, called *RC_FR* and *RC_RR*, respectively, to define the more valuable one in terms of reuse.
- Step 4: The manager compares *RC_FR* and *RC_RR* and starts the transfer if *RC_FR* is larger than *RC_RR*.

The CDDE scheme is an algorithm that allows in-DRAM caches to operate very carefully to maximize hit ratios, but does not consider power consumption due to mass transfer. Therefore, we propose a new in-DRAM cache management scheme that considers power consumption.

### 4.2. Power-Aware in-DRAM Cache Management Algorithm

Although the operation of the in-DRAM cache increases the power consumption as a result of massive data transfer, it also decreases the operating power owing to the reduced capacitance of the bit-line or shortened signal line between the core and I/O pads. These facts provide us an opportunity to compensate for the increase in transfer power. In other words, if the hit rate of the in-DRAM cache is sufficiently high enough to compensate for the increased transfer power, the overall power of the DRAM device can be maintained constant. In this work, we define several parameters. $P_{N}$ and $\alpha P_{N}$ represent the amounts of power consumed to access the normal DRAM and in-DRAM cache, respectively. Furthermore, we define the transfer power as $P_{M}$ and the hit ratio of the in-DRAM cache as *HR*. Along with the defined parameters, the total DRAM access energy over time of any activation count ($C_{A}$) is calculated as Equation (1).

(1) $$E_{acc}=\left\{P_{N}\times tRC\left(1-HR\right)+\alpha P_{N}\times tRC\left(HR\right)\right\}\times C_{A}$$

Equation (1) indicates that, as the hit rate of the in-DRAM cache increases, the overall access energy decreases. We will fill the reduced energy with transfer energy.

(2) $$E_{tran}=P_{T}\times T_{T}$$

The transfer energy is calculated as shown in Equation (2), where $P_{T}$ represents the transfer power consumed when the rows of all the ranks and banks are migrated. In addition, the $T_{T}$ indicates the time needed for a data transfer.

In this paper, we limit the total energy of the proposed scheme to be less than that of normal DRAM devices. Finally, Equation (3) shows the limiting condition.

(3) $$E_{acc}+E_{tran}<P_{N}\times tRC\times C_{A}$$

From Equations (1)–(3), we conclude that the transfer counts are limited, as shown in Equation (4).

(4) $$T_{T}<\frac{P_{N}\times tRC\times C_{A}\left(1-\alpha \right)HR}{P_{T}}$$

In Equation (4), all the parameters except *HR* of the right terms are predefined design parameters. Therefore, if the proposed scheme can monitor *HR* in real time, the available $T_{T}$ can be calculated periodically. The in-DRAM cache manager in the DRAM controller controls the *T*${}_{1}$ according to Equation (4).

**Figure 8:**
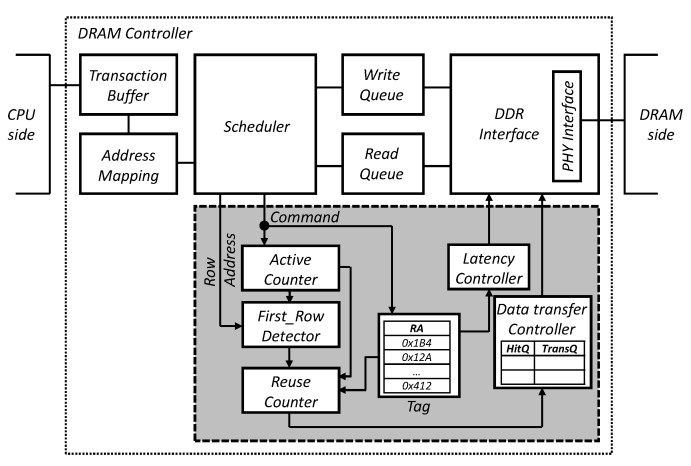
Implementations of in-DRAM cache manager on the DRAM controller.

Figure 8 shows the hardware implementation of the proposed scheme. The shaded part—the in-DRAM cache manager—must be added to the normal DRAM controller. The manager controls the timing constraints, such as *tRCD*, *tRP*, *tAA*, *tWR*, and *tRAS* when the addresses of the issued commands are included in the tag. The active counter identifies the four stages of CDDE, and the first row detector determines the most frequently accessed row address. Finding the *First_Row* is done in real time whenever an active row address is entered. The first row detector has as many counters as the number of bits in a row address. For example, if a row address is configured from 0 to 15, there will be a total of 16 counters. Therefore, when a row address is input, only the counters of bits corresponding to 1 out of the 16 bits are incremented by 1. At the end of Step 1 of the CDDE algorithm, the first row detector compares the total number of active inputs and the number of 1’s in each bit during step 1, and sets only the row address bits that are more than half of the active counts to 1. Finally, it returns *First_Row* consisting only of bits defined as 1 out of 16 bits. The reuse counter has two registers, one for storing the address of *First_Row* and the other for storing the row address to be replaced. In addition, it has a counter for each register, which increments each counter whenever a row address equal to the value of each register is input. Finally, it defines the more valuable row address in terms of hit rate with the counter output. Our proposed approach is applicable regardless of whether it is an open- or closed-page policy. In other words, the DRAM controllers using an open-page policy do not send multiple active commands continuously for a single row address. However, due to the specification of DRAM which requires only one row address to be activated in one bank, even if the open-page policy is used, there is a high possibility of accessing the same row address discontinuously. The data transfer controller contains a hit history queue (*HitQ*) and a transfer history queue (*TransQ*). It finally determines whether or not to execute a transfer according to the power-aware management algorithm.

Our proposed in-DRAM cache structure consists only of tags and data. This is because it can minimize tag access time, which is one of the most important factors of in-DRAM cache. Secondly, because it is not a multilevel structure like a typical cache, the tags do not need bits to store various information. In addition, our proposed in-DRAM cache operates in a write-through manner, minimizing the complexity of the cache itself and eliminating the latency penalty.

The biggest overhead in the in-DRAM cache manager is a tag that occupies from 1.125 KB to 4.5 KB. We used the CATTI tool to calculate its area and leakage power [22]. According to the CACTI tool, for a 32 nm technology, the tag requires 0.05 mm^2^ and consumes 1.2 mW standby leakage power. In addition, the time overhead of the tag is expected to be 2 ns, which can be minimized because the tag does not have any special information other than the row address and operates in a direct-mapped manner. Since the *HitQ* and *TransQ* each consist of 64 entries, we assumed that the area or time overhead could be ignored. The size of the tag may vary depending on the size of the in-DRAM cache. In contrast, the size of the *HitQ* and *TransQ* does not depend on the capacity of the in-DRAM cache, which is one of the design parameters.

## 5. Experimental Results and Discussion

In this paper, we have proposed two new in-DRAM cache management techniques. The ultimate goal of the both is to reduce DRAM latency by achieving maximum in-DRAM cache efficiency within a given energy budget. To evaluate the performance of the proposed techniques, we modeled a computing system including various 3D-stacked DRAM architectures using *gem5* and *DRAMSim2*, a modular platform for computer system architectures [23,24]. Table 1 shows the system and DRAM configurations used in the system simulation of this paper. The cache block size of 256 KB is equal to the total data size contained in a row address of all ranks and banks in the 3D-stacked DRAM. The tag for the in-DRAM cache is implemented in the DRAM controller with a direct-mapped manner by SRAM. We verify the effectiveness of the proposed schemes for various workloads of the *PARSEC* benchmark suite consisting of multi-threaded programs [25]. Table 2 summarizes the timing constraints for the normal DRAM and in-DRAM cache, where the *tAA* and *tWR* of the in-DRAM cache are only applied to the cache-bank architecture.

**Figure 9:**
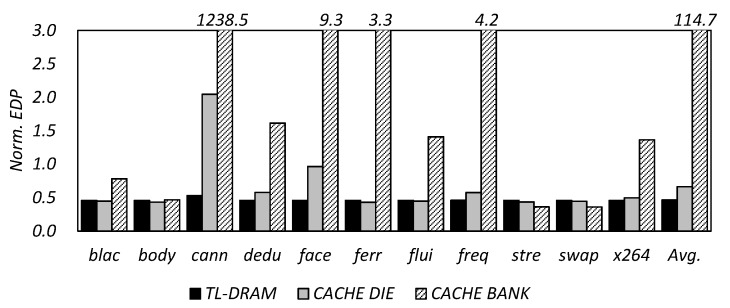
Normalized energy delay product (EDP) results for the TL-DRAM, cache die, and cache bank architecture which are managed by conventional FIFO cache management.

Figure 9 shows the energy delay product (EDP) results for the TL-DRAM, cache die, and cache bank architectures, which are managed by the conventional FIFO cache management (In this paper, all experimental results are normalized for a typical 3D-stacked DRAM without an in-DRAM cache.). As shown in Figure 9, TL-DRAM, which requires low transfer latency and power, has an average of 54% improvement in EDP across all workloads, even when using a conventional cache management scheme. However, for the cache die and cache bank, EDP increases by 2 and 1239 times, respectively, when the most memory-intensive workload *canneal* is running. That is, if the data locality of the workload is low, data transfer between the cache and the DRAM is more frequent and energy consumption due to the transfer becomes more serious. In particular, such a phenomenon is exacerbated in a cache bank-like structure having a small cache capacity. These results show that typical cache management schemes are not suitable for cache die and cache bank structures, although they may be appropriate for TL-DRAM, and require new algorithms for them.

**Figure 10:**
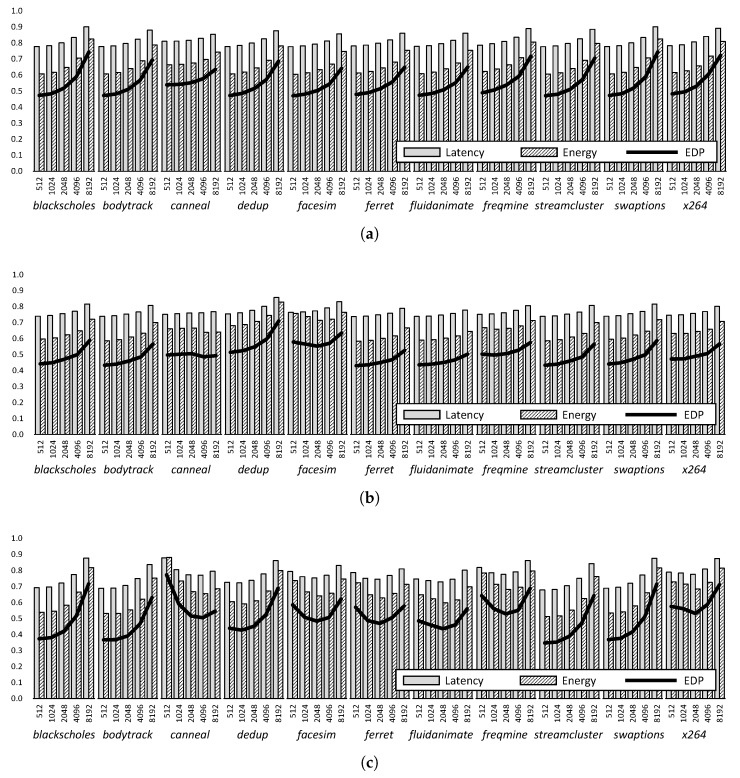
Normalized latency, energy, and EDP of TL-DRAM (**a**), cache die (**b**), and cache bank (**c**) structures for various unit cycles (*T*${}_{1}$) with CDDE.

To evaluate the effectiveness of the CDDE scheme, we experimented with the latency, energy, and EDP performance of 3D-stacked DRAMs with the TL-DRAM, cache die, and cache bank structures for various transfer cycles (*T*${}_{1}$), and Figure 10 shows the results. As shown in Figure 10, TL-DRAM exhibits better latency and EDP performance as the *T*${}_{1}$ is smaller, but the cache die and cache bank structure have an optimal *T*${}_{1}$ in terms of EDP depending on the properties of the workloads. Since the CDDE scheme helps prevent unnecessary data transfer between the in-DRAM cache and the DRAM, it can achieve better EDP performance over conventional cache management techniques. In addition, CDDE minimizes the EDP performance variation across the workloads compared to conventional management. When applying the conventional management, the difference of normalized EDP is shown to be 0.5 to 1238, according to the data locality (Figure 9). However, when CDDE is applied, it is shown to be 0.5 to 0.9. Despite the benefits of CDDE, it suffers from low EDP efficiency because it has to use a fixed *T*${}_{1}$, even though different *T*${}_{1}$s have to be applied to each application.

**Figure 11:**
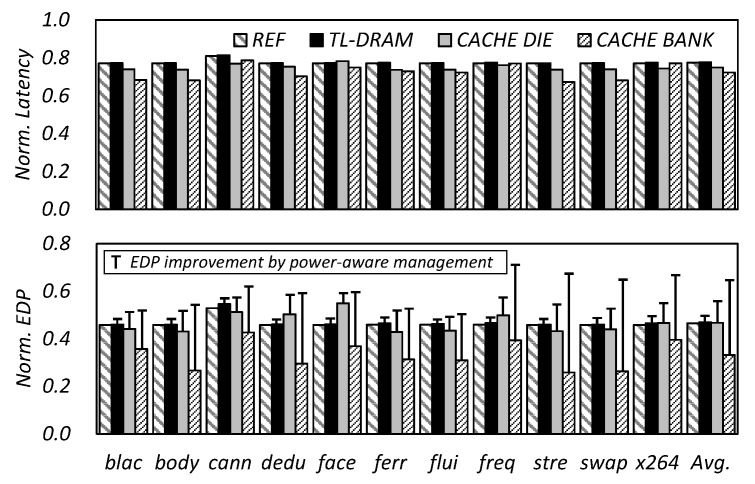
Normalized latency and EDP for the TL-DRAM, cache die, and cache bank architecture with the proposed algorithm. REFs are the latency and EDP results of TL-DRAM with conventional management.

In order to the overcome the drawbacks of CDDE, we implemented the power-aware in-DRAM cache management algorithm and evaluated its performance. Figure 11 shows that the average latency of 3D-stacked DRAMs improved by 22%, 25%, and 28% for the TL-DRAM, cache die, and cache bank, respectively, and EDP by 53%, 53%, and 67%, respectively. Applying the conventional cache management techniques to the in-DRAM cache, TL-DRAM had the best performance with 23% and 54% improvements in latency and EDP, respectively. However, when the proposed CDDE and power-aware management schemes were applied, the EDP of cache bank architecture showed 28% and 67% improvements in latency and EDP, respectively. This implies that although the TL-DRAM has low time and energy consumption for the data transfer, it is not sufficient to improve DRAM latency. In addition, adaptive management techniques, such as CDDE and power-aware which were proposed in this paper, can more effectively reduce DRAM latency in a structure that can basically maximize latency improvement, like cache die and cache bank.

## 6. Conclusions

Despite the recent introduction of various in-DRAM cache architectures, there was a lack of interest in how to manage them. In this paper, we studied how to derive optimal EDP by maximizing the hit ratio of In-DRAM cache and reducing power consumption due to data transfer. As a result, we achieved an improved EDP of 3D-stacked DRAM up to 67% compared to the conventional cache management scheme. Typical cache management techniques have several limitations when applied to the in-DRAM cache, and the effect depends on the architecture. However, the approach proposed in this paper demonstrates consistent improvements across all architectures.

## Figures and Tables

**Table 1:** System and dynamic random access memory (DRAM) configurations.

CPU Frequency	2 GHz
DRAM Types	DDR3 1600 (800 MHz)
DRAM Capacity	2 GB
in-DRAM Cache Capacity	TL-DRAM: 256 MB Cache-die: 512 MB Cache-bank: 128 MB
Cache Block Size	256 KB
Tag Size (DRAM controller)	TL-DRAM: 2.25 KB Cache-die: 4.5 KB Cache-bank: 1.125 KB
Row Buffer Policy	Adaptive Open Page
DRAM cells per a bit line	512 (DRAM) 64 (in-DRAM cache)
DRAM cells per a word line	1024
Refresh Rate	64 ms
Bit line array structure	Open bit-line
Transfer time per a row	128 * tCCD (5 ns) = 640 ns

**Table 2:** Timing constraints of the normal DRAM and the in-DRAM cache. ACT–activate, PRE–pre-charge, RD–read, WR–write.

Paramter	Symbol	Normal DRAM	in-DRAM Cache
Clock cycle	*tCK*	1.25 ns	1.25 ns
ACT to internal RD or WR delay	*tRCD*	13.75 ns	8.75 ns
PRE command period	*tRP*	13.75 ns	8.75 ns
ACT-to-PRE command period	*tRAS*	35.0 ns	15.0 ns
ACT-to-ACT command period	*tRC*	48.75 ns	23.75 ns
Internal RD command to data	*tAA*	13.75 ns	8.75 ns
Write recovery time	*tWR*	15.0 ns	10.0 ns

## Abbreviations

The following abbreviations are used in this manuscript:

DRAM	dynamic random access memory
OLTP	on-line transaction processing
TSV	through silicon via
HBM	high-bandwidth memory
HMC	hybrid memory cube
SRAM	static random access memory
CDDE	critical data detection and evaluation
LRU	least recently used
FIFO	first-in first-out
EDP	energy delay product
